# Birds in arid regions have depauperate louse communities: Climate change implications?

**DOI:** 10.1002/ece3.70280

**Published:** 2024-09-12

**Authors:** Sarah E. Bush, Matthew M. Waller, Kyle M. Davis, Sonora F. Clayton, Dale H. Clayton

**Affiliations:** ^1^ School of Biological Science University of Utah Salt Lake City Utah USA

**Keywords:** desiccation, host–parasite ecology, humidity, lice, new host records, parasite diversity, Passeriformes, Phthiraptera

## Abstract

Environmental factors such as temperature and humidity influence the distribution of free‐living organisms. As climates change, the distributions of these organisms change along with their associated parasites, mutualists and commensals. Less studied, however, is the possibility that environmental conditions may directly influence the distribution of these symbionts even if the hosts are able to persist in altered environments. Here, we investigate the diversity of parasitic lice (Insecta: Phthiraptera) on birds in arid Utah compared to the humid Bahamas. We quantified the parasite loads of 500 birds. We found that the prevalence, abundance and richness of lice was considerably lower among birds in Utah, compared to the Bahamas, despite sampling greater host taxonomic richness in Utah. Our data suggest that as climates change, birds in arid regions will have less diverse louse communities over time, potentially relieving birds of some of the cost of controlling these ectoparasites. Conversely, birds in more humid regions will see an increase in louse diversity, which may require them to invest more time and energy in anti‐parasite defense. Additional research with other ectoparasites of birds and mammals across different environmental conditions is needed to more fully understand how climate change may reshape parasite communities, and how these changes could influence their hosts.

## Graphical abstract

Environmental factors are known to affect the distribution of free‐living organisms. We surveyed parasitic lice (Insecta: Phthiraptera) of 500 birds and found that parasite diversity is also influenced by the environment. We found that birds in arid Utah had substantially lower louse diversity (prevalence, abundance, and richness) than birds in humid, subtropical, Bahamas.

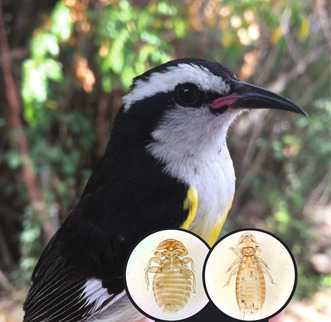

## 1 INTRODUCTION

Climate change increases the risk of extinction by altering habitats faster than species can adapt (Bellard et al., 2012; Thomas et al., 2004). Predicted effects of climate change apply not only to free‐living organisms, but also to species that live in close association with them, such as parasites, mutualists, and commensals (Dunn et al., 2009; Koh et al., 2004; Wood et al., 2023). In the case of parasites, climate change may reduce diversity in at least three ways. First, the specificity of many parasites puts them at risk of coextinction with hosts (Carlson et al., 2017). Second, a reduction in host population size may fall below the minimum threshold required to support parasite populations, even in the absence of host extinction (Bush et al., 2013). Third, climate change may increase the likelihood of parasite extinction by altering environmental parameters, such as the temperature or humidity, beyond levels that parasites can tolerate (Cizauskas et al., 2017).

**FIGURE 1:**
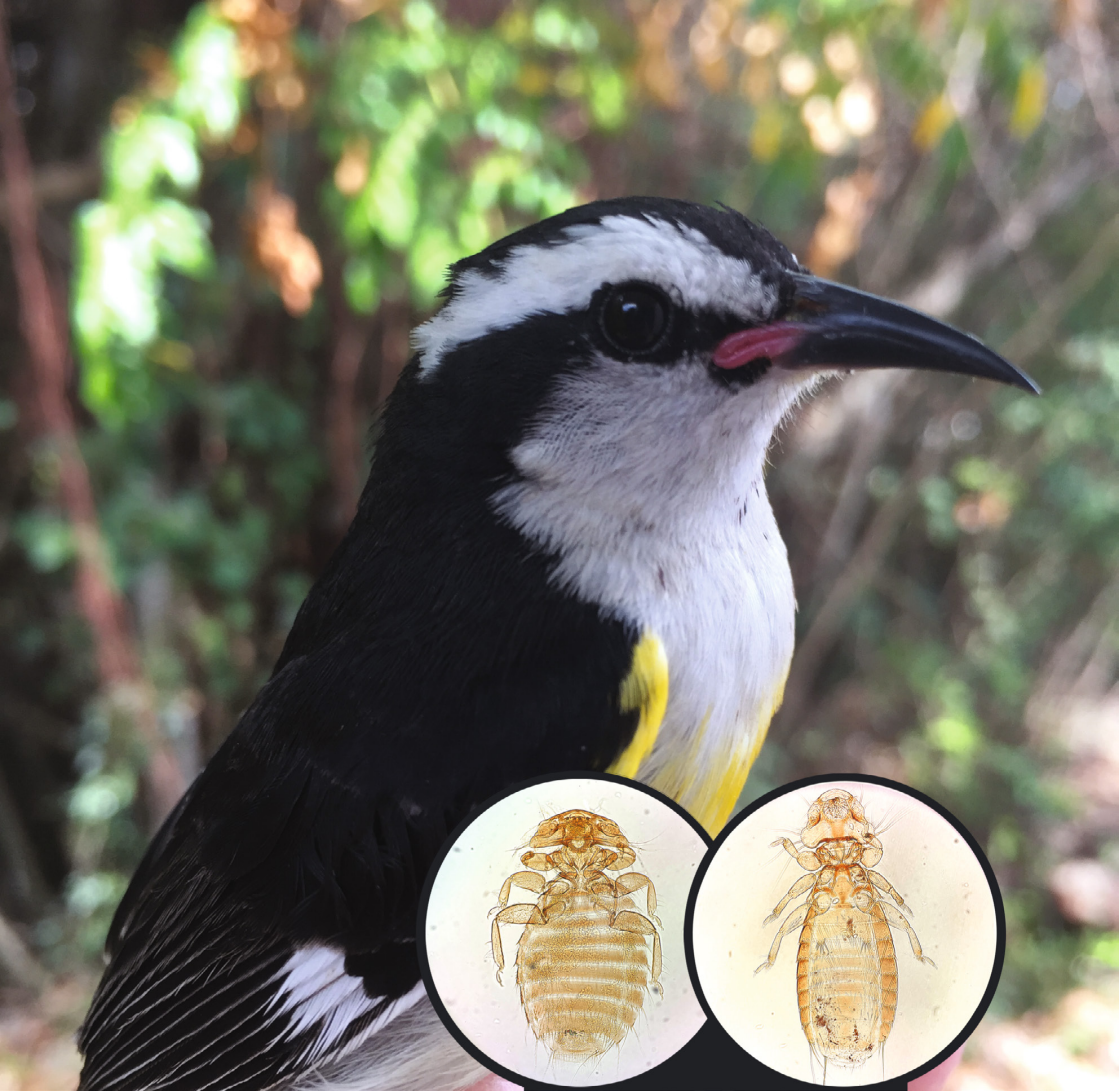
Birds, such as this bananaquit (*Coereba flaveola*), are commonly infested by parasitic lice that feed on feathers, dead skin and, in some cases, blood. (Insets of lice, left: *Machaerilaemus* sp.; right: *Myrsidea coerebicola*). Photos by SEB.

Ectoparasites may be particularly susceptible to the third option because they are generally more exposed to ambient conditions than endoparasites (Castaño‐Vázquez et al., 2022; Merino & Potti, 1996; Wiles et al., 2000). For example, fleas and ticks have free‐living stages off the host, and their survival and development are known to be affected by ambient temperature and humidity (Heeb et al., 2000; Krasnov et al., 2001; Ogden et al., 2021; Sáez‐Ventura et al., 2022). Permanent ectoparasites, which spend their entire life cycle on the body of the host, are exposed to ambient environmental conditions throughout their lives (Carrillo et al., 2007; Clayton et al., 2015). Bird lice (Insecta: Phthiraptera) are permanent ectoparasites that feed on feathers, dead skin and, in some cases, blood (Marshall, 1981) (Figure 1). Proximity to the host's body provides warmth that protects lice from extreme temperatures (Rothschild & Clay, 1952), but plumage does not provide a buffer against low ambient humidity (Moyer et al., 2002). Most bird lice have a water‐vapor uptake system that extracts moisture from the atmosphere. However, this system works efficiently only when atmospheric relative humidity is relatively high. (Rudolph, 1983).

Moyer et al. (2002) demonstrated that populations of lice on pigeons housed at low humidity do not survive well, if at all. Moreover, comparisons of lice on different species of Columbiformes (pigeons and doves) across different regions of the world revealed a positive relationship between louse prevalence and ambient humidity (Clayton et al., 2010; Moyer & Clayton, 2004). For example, lice were found on fewer than 3% of Columbiformes in the Sonoran Desert of Arizona, whereas 92% and 100% of Columbiformes in Philippine and Peruvian rainforests, respectively, had lice (Clayton et al., 2015). Similarly, a recent study of the American kestrel (Falconiformes: *Falco sparverius*) showed that the prevalence and abundance of lice are lower on kestrels living in arid regions of the United States (Utah), compared to kestrels living in the more humid subtropics (Bahamas) (Bush et al., 2023).

Despite the preponderance of lice in humid conditions in these studies, other studies of Passeriform birds show that some genera of lice can persist in arid environments. Carrillo et al. (2007) reported high prevalences of two genera of lice (*Brueelia* and *Philopterus*) on trumpeter finches (*Bucanetes githagineus*) in an arid region of Spain. A third genus of louse (*Myrsidea*) was absent, despite occurring on related species of finches (Price et al., 2003). Similarly, Bush et al. (2009) found that *Brueelia* and *Philopterus* occur on western scrub‐jays (*Aphelocoma californica*) in arid regions of the western United States. As in the case of trumpeter finches, the genus *Myrsidea* was absent, despite occurring on scrub‐jays in more humid regions. Bush et al. (2009) concluded that *Brueelia* and *Philopterus* tolerate arid conditions, while *Myrsidea* does not. Desiccation tolerance has been shown to have other ecological consequences for lice. Malenke et al. (2011) found that ambient humidity mediates the dynamics of interspecific competition in dove lice; dry conditions provide arid‐adapted species of lice with a refuge against competitively superior congeners.

Broader comparisons of ectoparasites on birds in humid versus arid regions of the world are needed to understand the role of environmental conditions on the louse communities of Passeriformes, which include more than half of all bird species. This paper provides the results of one such comparison. We compared the species richness, prevalence and abundance of louse communities on Passeriformes in climatologically diverse regions: a humid subtropical region (Bahamas) and an arid temperate region (northern Utah).

## 2 MATERIALS AND METHODS

We compared the diversity of ectoparasites on passeriform birds in a humid region (Bahamas) and an arid region (Utah, USA). More specifically, the humid location was San Salvador Island, Bahamas (24°N, 74°30′ W), with a mean annual humidity of 76.6% (driest month 74.2%, wettest month 79.2%), and a mean annual temperature of 26.0°C (coldest month 23.3°C, warmest month 28.6°C) (weatherandclimate.com). The arid location was Summit County, Utah, USA (41°01′ N,111°18°W), with a mean annual humidity of 56.5% (driest month 37.8°C with 4 months <40%, wettest month 81.4%) and a mean annual temperature of 11.0°C (coldest month −2.0°C, warmest month 25.3°C) (weatherandclimate.com). Fieldwork in the Bahamas was carried out March–April 2019; fieldwork in Utah was done May–July, 2021. Birds in both locations were captured with mist‐nets and subjected to “dust‐ruffling”, which accurately quantifies lice on passeriform birds (Koop & Clayton, 2013).

Freshly captured birds were removed from nets and isolated in single‐use paper bags stapled shut. The bags prevented mixing of parasites between birds, while also helping to keep the birds' calm. Each bird was removed from its bag and dust‐ruffled, during which the bird was held in one hand over a cafeteria tray lined with clean, white paper. Over the course of about 1 min, the other hand was used to work about a teaspoon of flea powder into the plumage of the wings, tail, keel, vent, back, head, and neck. Care was taken to avoid getting dust in the bird's nostrils or eyes. The dust was a pyrethrin‐based powder containing about 1.0% pyrethrins and 0.1% piperonyl butoxide, which acts as a synergist. Birds were held for another 2 min to allow the powder to take effect. Feather tracts were then ruffled for a total of 3 min with the fingers of the free hand. Ectoparasites falling onto the paper were located under a 4× jeweler's headset and transferred to a vial of 95% ethyl alcohol with a fine‐tipped brush. Three‐minute ruffling bouts were repeated until no additional parasites were recovered. Prior to release, each bird was weighed, measured, and marked with a numbered metal band, which allowed us to avoid re‐processing any birds recaptured during the study.

Most of the ectoparasites collected in this study were lice. Generic level identification of lice was by examination of unmounted specimens under a dissecting microscope and reference to generic‐level keys (Price et al., 2003) and the extensive reference collection in the Price Institute of Parasite Research (https://darwin.biology.utah.edu/PIPeRX.html). Species‐level identification of lice was based on published descriptions and host association data. In a few cases (e.g. Figure 1), lice were identified through detailed examination of slide‐mounted material. However, we chose to freeze most of the lice collected in this study for future molecular work, rather than mounting them on microscope slides.

Hippoboscid flies (Insecta: Diptera) were seen on occasion, but many of these escaped from birds as they struggled in the net and could thus not be identified. No fleas, bugs, or ticks were observed. Feather mites were frequently observed, but were not identified or quantified as part of this study. Voucher specimens and micrographs of all taxa of lice were deposited in the Price Institute of Parasite Research, University of Utah; voucher images are available at GBIF.org and Ecdysis.org.

We calculated three measures of parasite infestation: “prevalence” (percent of birds infested with parasites); “mean abundance” (mean number of parasites on individual birds); and “richness” (number of parasite taxa on birds) (Bush et al., 1997). We measured these parameters for a study total of 500 birds (26 species in each location).

Handling of birds in the Bahamas was performed in accordance with a 2019 permit from the Bahamas Environment, Science and Technology Commission (Ministry of the Environment), as well as the Institutional Animal Care and Use Committee of the University of Utah (IACUC protocol 17‐03012). Handling of birds in Utah was performed in accordance with a Utah Certificate of Registration (5COLL3669), U.S. Federal banding permits 21,384 and 24,077, and the Institutional Animal Care and Use Committee of the University of Utah (IACUC protocol 20–10,007).

### 2.1 Statistical analyses

For analyses concerning the entire dataset, louse prevalence between the Bahamas and Utah were compared with a simple Fisher's exact test. For between site comparisons of louse abundance, we took host body mass into account because parasite abundance across diverse groups of host species is usually correlated with host body mass (Clayton et al., 2015; Poulin, 2011). We estimated the relationship between geographic location, host body mass, and the interaction between location and body mass on louse abundance using a generalized regression with a zero‐inflated negative binomial distribution; this distribution was chosen because lice are often over‐dispersed among individual birds in natural populations (Clayton et al., 2015); indeed, we recovered no lice from a majority (71.6%) of the 500 birds we sampled (Tables 1 and 2).

**TABLE 1:** Bird species surveyed for lice on San Salvador Island, Bahamas.

Family	Species	Mean mass (g)	# Birds total	# Birds infested	# Lice (range)
Cardinalidae (Cardinals and Allies)	***Passerina cyanea***	**14.4**	**22**	**2**	**4**
Mimidae (Mockingbirds and Thrashers)	***Dumetella carolinensis***	**35.8**	**15**	**6**	**5–47**
	*Margarops fuscatus*	92.3	3	3	55–157
	*Mimus gundlachii*	64.7	9	7	31–210
	*Mimus polyglottos*	45.3	3	3	6–58
Parulidae (New World Warblers)	*Geothlypis trichas*	8.3	2	2	8–11
	*Helmitheros vermivorum*	12.8	3	0	–
	*Mniotilta varia*	8.5	5	0	–
	*Parkesia noveboracensis*	15.8	2	0	–
	*Protonotaria citrea*	13.5	2	1	13
	***Seiurus aurocapilla***	**20.8**	**17**	**6**	**2–14**
	*Setophaga americana*	7.0	1	1	1
	*Setophaga caerulescens*	10.2	3	0	–
	*Setophaga coronata*	9.8	2	1	3
	***Setophaga discolor***	**7.4**	**13**	**4**	**2–18**
	*Setophaga dominica*	8.5	1	0	–
	*Setophaga palmarum*	10.0	3	3	3–22
	*Setophaga petechia*	8.9	10	8	2–37
	*Setophaga ruticilla*	7.3	6	0	–
	*Setophaga tigrina*	9.5	1	1	7
Thraupidae (Tanagers and Allies)	***Coereba flaveola***	**10.8**	**65**	**61**	**1–40**
	***Melanospiza bicolor***	**10.2**	**19**	**6**	**3–40**
Tyrannidae (Tyrant Flycatchers)	*Myiarchus sagrae*	12.0	1	0	–
Vireonidae (Vireos and Allies)	*Vireo altiloquus*	15.9	6	3	3–20
	***Vireo crassirostris***	**13.9**	**21**	**4**	**1–40**
	*Vireo griseus*	11.3	6	3	18–140
	Total # individual birds		241	127 (52.7%)	
	Total # bird species		26 spp	20 spp	

*Note*: Boldface entries are species with at least 12 individuals sampled for lice.

**TABLE 2:** Bird species surveyed for lice in Summit County, Utah.

Family	Species	Mean mass (g)	# Birds total	# Birds infested	# Lice (range)
Cardinalidae (Cardinals and Allies)	*Passerina amoena*	15.5	2	0	–
	*Pheucticus melanocephalus*	45.6	9	0	–
	*Piranga ludoviciana*	29.7	7	0	–
Paridae (Tits, Chickadees, and Titmice)	*Poecile atricapillus*	10.7	8	0	–
Parulidae (New World Warblers)	***Geothlypis tolmiei***	**10.7**	**31**	**1**	**5**
	***Leiothlypis celata***	**9.5**	**56**	**7**	**1–3**
	***Leiothlypis virginiae***	**10.3**	**19**	**1**	**1**
	*Setophaga coronata*	12.0	1	0	–
	*Setophaga petechia*	8.0	1	0	–
Passerellidae (New World Sparrows)	*Junco hyemalis*	18.0	1	0	–
	*Melospiza lincolnii*	17.7	3	0	–
	*Melospiza melodia*	21.0	1	0	–
	***Pipilo chlorurus***	**28.7**	**25**	**3**	**1–14**
	***Pipilo maculatus***	**35.9**	**46**	**3**	**1–7**
	*Spizella breweri*	16.7	3	0	–
	*Spizella passerina*	13.8	4	0	–
Regulidae (Kinglets)	*Corthylio calendula*	6.0	1	0	–
Troglodytidae (Wrens)	*Troglodytes aedon*	9.0	1	0	–
Turdidae (Thrushes and Allies)	*Catharus guttatus*	28.5	2	0	–
	***Turdus migratorius***	**77.6**	**14**	**0**	–
Tyrannidae (Tyrant Flycatchers)	*Contopus cooperi*	34.0	1	0	–
	*Empidonax difficilis*	11.0	1	0	–
	*Empidonax hammondii*	12.0	1	1	1
	***Empidonax oberholseri***	**11.3**	**14**	**0**	–
Vireonidae (Vireos and Allies)	*Vireo gilvus*	10.3	6	0	–
	*Vireo plumbeus*	17.0	1	0	–
	Total # of individual birds		259	16 (6.2%)	
	Total # of bird species		26 spp	6 spp	

*Note*: Boldface entries are species with at least 12 individuals sampled for lice.

In addition to our overall analyses, we compared the diversity of lice on a more restricted data set consisting of seven species of birds from each location with at least 12 individuals sampled for lice. This criterion is based on Bush et al.'s (2013) demonstration that at least 12 individuals need to be sampled to achieve a 90% probability of detecting all species of ectoparasites on a given species of host. Seven species of birds from each geographic location were sampled in sufficient numbers to meet these criteria (boldface taxa in Tables 1 and 2). For this dataset we analyzed the mean louse generic richness and mean louse prevalence with standard tests (Wilcoxon rank sum and one‐way ANOVA, respectively). The effects on louse abundance of geographic location, host mass, and the interaction between location and host mass were estimated using a generalized regression with a negative binomial distribution; this distribution was chosen because louse abundance was over‐dispersed.

All analyses were conducted in JMP®v.16, SAS Institute Inc., Cary, NC, USA, 1989–2021.

## 3 RESULTS

We captured and dust‐ruffled 500 birds, 241 in the Bahamas and 259 in northern Utah. Bahama birds represented 26 species from 15 genera and 6 families (Table 1). Utah birds represented 26 species from 18 genera and 9 families (Table 2).

### 3.1 Lice on Bahama birds

Lice were found on 127 of the 241 Bahama birds, for an overall prevalence of 52.7% (Table 1). In total, we documented 33 host associations involving seven genera of lice (Table 3). Nineteen of the host associations are new records (Gustafsson & Bush, 2017; Price et al., 2003). Future taxonomic work is likely to reveal that some of these associations involve undescribed species of lice.

**TABLE 3:** Host records of lice found on San Salvador Island, Bahamas.

Host family	Host species (*n*)	Lice^a^, ^b^	# birds infested	Intensity (range)	Voucher catalog #
Cardinalidae (Cardinals and Allies)	***Passerina cyanea* (22)**	***Machaerilaemus* sp.** ^a^	**2**	**4**	PIPR050065
Mimidae (Mockingbirds and Thrashers)	***Dumetella carolinensis* (15)**	***Guimaraesiella brunneinucha*** ^c^	**2**	**6–11**	PIPR050049
		***Myrsidea* sp.** ^a^	**5**	**5–47**	PIPR050050
	*Margarops fuscatus* (3)	*Brueelia* sp.^a^	1	1	PIPR050056
		*Myrsidea* sp.^a^	3	49–141	PIPR050058
		*Philopterus* sp.^a^	3	6–16	PIPR050057
	*Mimus gundlachii* (9)	*Myrsidea* sp.^a^	7	48–210	PIPR050059
		*Philopterus* sp.^a^	1	1	PIPR050060
	*Mimus polyglottos* (3)	*Myrsidea* sp.^d^	3	6–58	PIPR050041
Parulidae (New World Warblers)	*Geothlypis trichas* (2)	*Myrsidea* sp.^a^	2	7–11	PIPR050055
		*Brueelia‐complex* sp.^c^, ^e^	1	1	PIPR050054
	*Protonotaria citrea* (2)	*Ricinus pallens* ^f^	1	13	PIPR050082
	***Seiurus aurocapilla* (17)**	***Brueelia‐*complex sp.** ^c^, ^e^	**1**	**4**	PIPR050051
		***Myrsidea* sp.** ^a^	**5**	**2–14**	PIPR050066
	*Setophaga americana* (1)	*Myrsidea* sp.^a^	1	1	PIPR050064
	*Setophaga coronata* (2)	*Ricinus dendroicae* ^g^	1	3	PIPR050061
	***Setophaga discolor* (13)**	***Myrsidea* sp.** ^a^	**1**	**1**	PIPR050068
		***Ricinus dendroicae*** ^g^		**2–18**	PIPR050067
	*Setophaga palmarum* (3)	*Ricinus dendroicae* ^g^	3	3–22	PIPR050069
	*Setophaga petechia* (10)	*Ricinus dendroicae* ^g^	6	1–15	PIPR050062
		*Myrsidea ridulosa* ^h^	8	1–31	PIPR050083
	*Setophaga tigrina* (1)	*Myrsidea* sp.^a^	1	7	PIPR050084
Thraupidae (Tanagers and Allies)	***Coereba flaveola* (65)**	***Brueelia phasmasoma*** ^c^	**41**	**1–11**	PIPR050045
		***Machaerilaemus* sp.** ^a^	**10**	**1–8**	PIPR050043
		***Myrsidea coerebicola*** ^i^	**53**	**1–36**	PIPR050044
	***Melanospiza bicolor* (19)**	***Myrsidea* sp.** ^a^	**6**	**3–40**	PIPR050070
Vireonidae (Vireos and Allies)	*Vireo altiloquus* (6)	*Ricinus* sp.^a^	3	3–20	PIPR050073
		*Philopterus* sp.^a^	1	10	PIPR050072
		*Brueelia‐complex* sp.^a^, ^c^	1	1	PIPR050071
	***Vireo crassirostris* (21)**	***Myrsidea* sp.** ^a^	**2**	**1–40**	PIPR050077
		***Ricinus vireoensis*** ^g^	**2**	**18–19**	PIPR050074
	*Vireo griseus* (6)	*Menacanthus curuccae* ^j^	2	18–140	PIPR050076
		*Philopterus* sp.^a^	1	65	PIPR050075

*Note*: Boldface entries are species with at least 12 individuals sampled for lice.

^a^ New host record.

^b^ Lice identifications based on Price et al (2003) unless otherwise indicated.

^c^ Gustafsson and Bush (2017).

^d^ Unidentified *Myrsidea* sp. also noted from *Mimus polyglottos* in Pistone et al. (2021).

^e^ Unidentified *Brueelia*‐complex sp. also noted from *Geothlypis trichas* in Galloway et al. (2014), and *Seiurus aurocapilla* in Bush et al. (2016).

^f^ Kellogg (1899).

^g^ Nelson (1972).

^h^ Kellogg and Chapman (1899).

^i^ Klockenhoff and Schirmers (1976).

^j^ Price (1977).

### 3.2 Lice on Utah birds

Lice were found on 16 of the 259 Utah birds, for an overall prevalence of 6.2% (Table 2). In total, we documented six host associations involving two genera of lice (Table 4). Four of the host associations are new records (Price et al., 2003). Whether any of these records involve undescribed species of lice awaits future taxonomic work.

**TABLE 4:** Host records of lice found in Summit County, Utah.

Host family	Host species (*n*)	Lice^a^, ^b^	# Birds infested	Intensity (range)	Voucher catalog #
Parulidae (New World Warblers)	***Geothlypis tolmiei* (31)**	***Ricinus* sp.**	**1**	**5**	PIPR050007
	***Leiothlypis celata* (56)**	***Ricinus* sp.**	**7**	**1–3**	PIPR050001
	***Leiothlypis virginiae* (19)**	***Ricinus* sp.** ^a^	**1**	**1**	PIPR050005
Passerellidae (New World Sparrows)	***Pipilo chlorurus* (25)**	***Ricinus* sp.** ^a^	**4**	**1–14**	PIPR050039
	***Pipilo maculatus* (46)**	***Ricinus* sp.** ^a^	**3**	**1–7**	PIPR050012
Tyrannidae (Tyrant Flycatchers)	*Empidonax hammondii* (1)	*Philopterus* sp.^a^	1	1	PIPR050006

*Note*: Boldface entries are species with at least 12 individuals sampled for lice.

^a^ New host record.

^b^ Lice identifications based on Price et al. (2003).

### 3.3 Overall comparison of lice on Bahama and Utah birds

We sampled 26 species of Passeriformes in the Bahamas and 26 species of Passeriformes in Utah. The richness of higher bird taxa in Utah was greater than in the Bahamas, with 20% more bird genera and 50% more bird families sampled in Utah (Table 5). Despite this difference, the ectoparasite community on Utah birds was less rich than that on Bahama birds. The mean number of parasite genera per host family, genus, and species were all significantly lower in Utah than the Bahamas (Table 5). The prevalence of lice on Utah birds was also significantly lower than on Bahama birds (Fisher's exact, *n* = 500, *p* < .0001).

**TABLE 5:** Comparison of the generic richness of lice from the Bahamas and Utah at different host taxonomic levels.

	Bahamas	Utah	Wilcoxon rank sum
**Total # lice genera**	**7**	**2**	
**Mean lice genera per host family**	**2.67**	**0.33**	*n* = 14, *z* = 2.3, ***p* = .017**
#host families sampled	6	9	
**Mean lice genera per host genus**	**1.53**	**0.22**	*n* = 33, *z* = 3.4, ***p* = .0008**
#host genera sampled	15	18	
**Mean lice genera per host species**	**1.27**	**0.23**	*n* = 52, *z* = 4.0, ***p* < .0001**
#host species sampled	26	26	

Parasite abundance is often correlated with host body mass, and the mean body mass of birds in our study differed by location. The mean (±SE) body mass of Bahama birds (17.05 ± 1.09 g) was significantly less than that of Utah birds (22.53 ± 1.05 g) (oneway ANOVA df = 1497; *F* = 13.00, *p* = .0003); thus, our analysis of factors influencing louse abundance included host location, host body mass, and the interaction between these main effects (see the “methods” section for more details).

**FIGURE 2:**
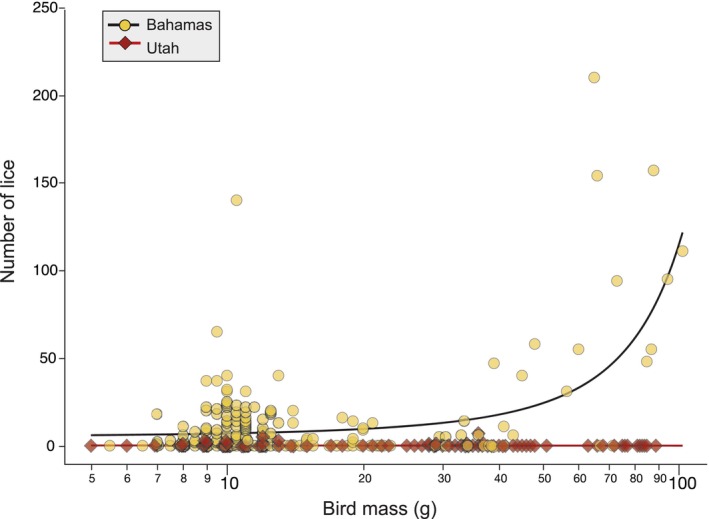
Relationship between host body mass and abundance of lice on Utah birds compared to Bahama birds. Lines are best fit based on a generalized regression model with a zero‐inflated negative binomial distribution (see results). The sample size for Utah was 257 birds, slightly less than the 259 birds in Table 2 because two Utah birds were not weighed.

The abundance of lice on Bahama birds (mean (±SE) 11.31 ± 1.66; *n* = 241) was significantly greater than that on Utah birds (0.17 ± 0.07; *n* = 257; the sample size is slightly less than shown in Table 2 because two Utah birds were not weighed; Wald *χ* ^2^ = 319.35, *p* < .001; Figure 2). Body mass alone was not significantly correlated with louse abundance (Wald *χ* ^2^ = 0.99, *p* = .32), but there was a significant interaction between location and body mass on louse abundance (Wald *χ* ^2^ = 7.84, *p* = .005). Louse abundance increased with host body mass in the Bahamas, but not in Utah, where even larger bodied birds were seldom infested.

### 3.4 Restricted comparison of lice on Bahama and Utah birds

**FIGURE 3:**
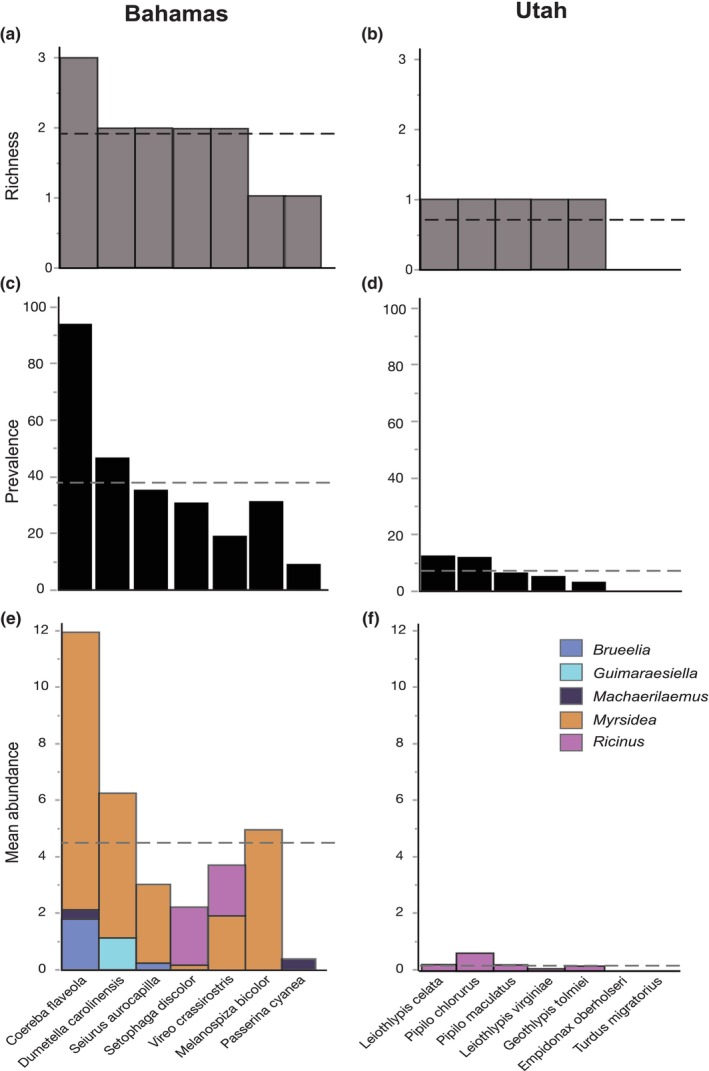
Generic richness (a, b), prevalence (c, d), and mean abundance (e, f) of lice on a restricted data set of the seven bird species with at least 12 individuals sampled for lice in each of two geographic regions. Host taxa (*x* axis) arranged in order of decreasing richness, then prevalence, within each region. Dashed lines are the mean of each measure per region.

We conducted additional analyses on a restricted dataset of birds consisting of seven bird species in the Bahamas and seven bird species in Utah in which at least 12 individuals were sampled. We analyzed the mean louse generic richness, prevalence, and abundance for each of these bird species (Figure 3).

Within this restricted dataset, five genera of lice were found in the Bahamas (*Brueelia*, *Guimaraesiella*, *Machaerilaemus*, *Myrsidea*, and *Ricinus*), compared to one genus (*Ricinus*) in Utah. The mean generic richness per host species was significantly higher in the Bahamas (1.88 genera; Figure 3a) than Utah (0.71 genera; Figure 3b) (Wilcoxon rank sum, *n* = 14, *z* = −2.63, *p =* .007). The mean (±SE) louse prevalence was also significantly higher in the Bahamas (38.0% ± 10.3%, *n* = 7; Figure 3c) than Utah (5.6% ± 1.9%, *n* = 7; Figure 3d) (oneway ANOVA, *F*_1,13_ = 9.5, *p* = .0095).

Mean (±SE) abundance was significantly greater in the Bahamas (4.65 ± 1.42, *n* = 7 spp; Figure 3e) than that in Utah (0.18 ± 0.08, *n* = 7 spp; Figure 3f) (Wald *χ* ^2^ = 14.8, *p* < .001). For the restricted dataset, neither mass, nor the interaction between location and mass, were significantly correlated with louse abundance (*p* ≥ .79).

## 4 DISCUSSION

Our data show that birds in arid Utah have much less diverse communities of lice than birds in the humid Bahamas. The prevalence of lice on birds in Utah was much less than in the Bahamas; overall, only 6.2% of Utah birds had lice, compared to 52.7% of Bahama birds (Tables 1 and 2). The generic richness of lice on birds in Utah was also much less than that in the Bahamas. Across all 26 species of birds in each location, only two genera of lice were found in Utah, compared to seven genera in the Bahamas. This difference was particularly striking, considering that the sampled host taxonomic diversity was greater in Utah than the Bahamas. The generic richness of lice was significantly lower in Utah than the Bahamas at all three levels of host classification: species, genus, and family (Table 5). We did not compare the species level richness of lice between the two locations because future taxonomic work is required to identify many of the lice to species, including probable new species.

The abundance of lice on Utah birds was also much lower, with a mean of 0.17 lice on Utah birds, compared to 11.31 lice on Bahama birds (Figure 2). This difference was apparent despite the fact that the sampled Utah birds were about 32% larger, on average, than Bahama birds (mean body mass: 22.53 and 17.05 g, respectively). While parasite abundance is often correlated with host size (Poulin, 2011), even the larger birds in Utah rarely harbored lice.

In addition to our overall analyses, we compared the diversity of lice on a more restricted data set consisting of seven species of birds from each location that were more intensively sampled (>12 individuals per species). For this restricted data set, the generic richness, prevalence, and mean abundance of lice were all much lower on Utah birds than Bahama birds (Figure 3).

The lower diversity of lice in Utah, compared to the Bahamas, is consistent with earlier comparative and experimental work showing that most bird lice do not fare well in arid environments. However, this pattern is restricted to lice that feed on feathers. Among the 259 birds we examined in Utah, only one feather feeding louse was found, a single individual of *Philopterus* sp. from *Empidonax hammondii*. All of the other lice recovered from birds in Utah were *Ricinus*, a genus that feeds exclusively on blood (Nelson, 1972). In Utah, *Ricinus* occurred on a total of 16 birds representing five species (Table 4). In the Bahamas, *Ricinus* occurred on a total of 17 birds representing seven species (Table 3). In short, the only genus of louse in our study with similar diversity in Utah and the Bahamas was the only genus with a liquid diet. All of the other genera of lice in our study feed primarily on feathers and dead skin (Clayton et al., 2015).

Rudolph (1983) measured rates of water‐uptake and water‐loss for 10 species of bird lice. The ability of lice to withstand arid conditions varied dramatically among species. He found that *Trinoton querquedulae*, which also feeds on blood, cannot extract water from the atmosphere. In contrast, two species of exclusively feather‐feeding lice (*Campanulotes compar* and *Columbicola columbae*) were able to maintain a water equilibrium at a relative humidity of just 43%. Both of these species commonly occur on pigeons (*Columba livia*) in Utah, although even these species are much more prevalent and abundant in more humid regions (Moyer et al., 2002). Studies by Carrillo et al. (2007) and Bush et al. (2009) both document the occurrence of lice in the genera *Brueelia* and *Philopterus* in arid regions; perhaps some species of lice in these genera are also able to balance water uptake and loss in very arid conditions.

In addition to differences in humidity, Utah and the Bahamas differ considerably in temperature. However, differences in temperature are unlikely to have contributed to the observed difference in diversity of lice in the Bahamas and Utah. Feathers buffer temperature near the skin, and lice experience consistently warm conditions (Clayton et al., 2015). By contrast, feathers do not buffer humidity near the skin (Moyer et al., 2002). When geographic regions with similar humidity are compared, birds in warm tropical and cool temperature zones do not differ appreciably in louse diversity. Clayton et al. (1992) reported no difference in the diversity of lice on birds in the Peruvian rainforest, compared to lice in Newfoundland, Canada, where the mean annual temperature is much lower (Wheeler & Threlfall, 1986). Notably, both of these locations are very humid (mean annual low relative humidity for each location >62%; weatherandclimate.com).

Current climate models predict changes in the humidity of geographic regions around the globe, with some areas becoming more arid, and other areas becoming more humid (Berg & McColl, 2021; Huang et al., 2016; Koutroulis, 2019). Our data suggest that regional changes in ambient humidity will influence the diversity and distribution of ectoparasites, such as lice. Feather‐feeding lice are less likely to persist in arid environments, compared to humid environments. Changing parasite distributions may also reciprocally affect the hosts. Birds in arid regions should experience release from parasitism by ectoparasites, meaning that they may not need to invest as much time and energy in anti‐parasite defenses, such as grooming behavior (Bush & Clayton, 2018). In contrast, birds living in regions that become more humid, may need to invest more time and energy keeping ectoparasites under control (Bush et al., 2023). In addition to overall changes in louse abundance correlated with humidity, our data suggest that the composition of louse communities will change. Blood‐feeding lice, such as *Ricinus* spp., are likely to persist even in arid environments. Since these lice interact with the immune system, immune responses may keep these parasites in check (Bush & Clayton, 2018; Owen et al., 2010; Tschirren & Richner, 2006; Vlček & Štefka, 2020).

By themselves, parasites are not likely to drive host populations to local extinction (Brian, 2023). Over macroevolutionary time, birds have amassed a large arsenal of defenses against parasites and pathogens (Clayton et al., 2010). However, the speed with which climate is changing poses new challenges, and the combination of increasing parasite pressure along with habitat change, habitat loss, and other environmental stressors may be overwhelming. Parasites, combined with environmental stressors, are known to have devastating effects on populations of fish (Gheorgiu et al., 2006), amphibians (Hof et al., 2011), and birds (Atkinson, 2023).

Lice and many other parasites and mutualists face coextinction with their hosts (Colwell et al., 2012; Dunn et al., 2009), as well as extinction in the face of habitat loss and climate change (Bush et al., 2013; Wood et al., 2023). Parasites are an enormous part of global biodiversity (Poulin, 2011) and are key parts of healthy ecosystems (Hudson et al., 2006). Parasites may also contribute to the evolution of host diversity (Betts et al., 2018; Clayton et al., 2015; Ebert & Fields, 2020). We are just beginning to understand what factors influence parasite persistence in a changing world (Carlson et al., 2020). Here, we have shown that the distribution of feather‐feeding lice is strongly influenced by environmental conditions. Future work with diverse ectoparasite communities across different host populations, regions, and environmental conditions are needed to understand how climate change is likely to impact other parasites and their hosts.

## AUTHOR CONTRIBUTIONS

**Sarah E. Bush:** Conceptualization (equal); data curation (equal); formal analysis (equal); funding acquisition (equal); investigation (equal); methodology (equal); project administration (equal); resources (equal); supervision (equal); visualization (equal); writing – original draft (equal); writing – review and editing (equal). **Matthew M. Waller:** Data curation (equal); formal analysis (equal); investigation (equal); visualization (equal); writing – original draft (supporting); writing – review and editing (supporting). **Kyle M. Davis:** Data curation (equal); formal analysis (supporting); investigation (equal); writing – original draft (supporting); writing – review and editing (supporting). **Sonora F. Clayton:** Investigation (supporting); writing – review and editing (supporting). **Dale H. Clayton:** Conceptualization (equal); data curation (supporting); formal analysis (equal); funding acquisition (equal); investigation (equal); methodology (equal); project administration (equal); resources (equal); supervision (equal); writing – original draft (equal); writing – review and editing (equal).

## CONFLICT OF INTEREST STATEMENT

The authors have no conflicts of interest to declare.

## Supporting information


Data S1.

## Data Availability

Original data are provided as Supporting Information. Voucher specimens have been deposited at the Price Institute of Parasite Research, University of Utah; images of vouchers are publicly available online at Ecdysis.org which is harvested by data aggregators like GBIF.org and iDigBio.org. The data that support the findings of this study are available from: https://datadryad.org/stash/share/WFaWTXudtflyQOVegtddo9KcYHf9HcGqnd0uypV22Aw.
